# Overexpression of SMYD3 Promotes Autosomal Dominant Polycystic Kidney Disease by Mediating Cell Proliferation and Genome Instability

**DOI:** 10.3390/biomedicines12030603

**Published:** 2024-03-07

**Authors:** Ewud Agborbesong, Julie Xia Zhou, Hongbing Zhang, Linda Xiaoyan Li, Peter C. Harris, James P. Calvet, Xiaogang Li

**Affiliations:** 1 Department of Internal Medicine, Mayo Clinic, Rochester, MN 55905, USA; 2 Department of Biochemistry and Molecular Biology, Mayo Clinic, Rochester, MN 55905, USA; 3 Department of Biochemistry and Molecular Biology, University of Kansas Medical Center, Kansas City, KS 66160, USA

**Keywords:** nephrology, ADPKD, epigenetics, SMYD3, genome instability

## Abstract

Autosomal dominant polycystic kidney disease (ADPKD) is the most common inherited kidney disorder worldwide and progresses to end-stage renal disease (ESRD). However, its precise mechanism is not fully understood. In recent years, epigenetic reprogramming has drawn increasing attention regarding its effect on cyst growth. However, considering the complexity of epigenetic mechanisms and the broad range of alterations of epigenetic components in ADPKD, identifying more specific epigenetic factors and understanding how they are mechanistically linked to promote cyst growth is relevant for the development of treatment for ADPKD. Here, we find that the histone methyltransferase SMYD3, which activates gene transcription via histone H3 lysine 4 trimethylation (H3K4me3), is upregulated in *PKD1* mutant mouse and human ADPKD kidneys. Genetic knockout of *SMYD3* in a *PKD1* knockout mouse model delayed cyst growth and improved kidney function compared with *PKD1* single knockout mouse kidneys. Immunostaining and Western blot assays indicated that SMYD3 regulated PKD1-associated signaling pathways associated with proliferation, apoptosis, and cell cycle effectors in *PKD1* mutant renal epithelial cells and tissues. In addition, we found that SMYD3 localized to the centrosome and regulated mitosis and cytokinesis via methylation of α-tubulin at lysine 40. In addition, SMYD3 regulated primary cilia assembly in *PKD1* mutant mouse kidneys. In summary, our results demonstrate that overexpression of SMYD3 contributes to cyst progression and suggests targeting SMYD3 as a potential therapeutic strategy for ADPKD.

## 1. Introduction

Autosomal dominant polycystic kidney disease (ADPKD) is the most common heritable renal disease, estimated at a prevalence of 1 in 500 to 1 in 1000 people worldwide [1]. It is genetically heterogeneous and has been linked to mutations in the *PKD1* and *PKD2* genes [2]. Mutations in the *PKD1* gene, which encodes the polycystin-1 (PC1) protein, account for approximately 85% of ADPKD cases, while the *PKD2* gene, which encodes the polycystin-2 (PC2) protein, accounts for approximately 15% of ADPKD cases [2]. Typically, the disease is characterized by the development of bilateral fluid-filled cysts in the kidneys that progressively enlarge and often lead to end-stage renal disease (ESRD). Based on the focal nature of these cysts [3], a “two-hit hypothesis” (a second molecular hit) is necessary for the development of ADPKD [4]. While there is some correlation between the *PKD1* gene mutation, the functional PC1 dosage, and ADPKD disease severity [5], clinical manifestations and the course of the disease are reportedly variable.

The pathogenesis underlying the ADPKD phenotypes is still unclear; however, various cellular aberrations associated with cystogenesis have been suggested, including centrosome aberrations [6]. The centrosome is the primary microtubule-organizing center in dividing eukaryotic cells [7]. Once during every cell cycle, centrosome duplication takes place, with a daughter centriole forming next to an existing mother centriole in a semiconservative manner [8]. Malfunctions in this process sometimes lead to centrosome amplification, a common feature of human cancers that can arise from diverse mechanisms such as failure in cell division, centrosome overduplication, and multipolar spindle division [9]. The presence of supernumerary centrosomes leads to the formation of multipolar mitotic spindles and cytokinesis failure, resulting in chromosome segregation, culminating in chromosomal instability [10,11,12]. In general, the presence of these abnormalities leads to cell death [13]. However, over time, a sub-population of cells acquire the ability to survive extra centrosomes and chromosome instability, and the accumulation of these abnormalities contributes to disease.

Centrosome amplification has been observed in the kidneys of patients and animal models of several types of cystic kidney diseases. Also, loss of the PC1 protein induced centrosome amplification in renal cells in vitro [6], and centrosome amplification was observed in kidneys of ADPKD patients and PKD2 transgenic mice in vivo [14]. The link between centrosome amplification and genomic instability has profound implications on the pathogenesis of ADPKD, since genomic instability has been suggested as a mechanism for the somatic mutation and emergence of the genotypic heterogeneity necessary for the development of cyst growth [6]. However, the identity of the factors and the molecular mechanisms involved in the regulation of centrosome integrity and subsequent genome instability in ADPKD remain unclear.

The importance of the centrosome in the cystogenic process is also underlined by the localization of most cystogenic proteins within the centrosome/basal body or the primary cilium [15,16]. The primary cilium is a highly specialized, microtubule-based sensory organelle that is anchored by the basal body which originates from the mother centriole, and projects from the surface of most mammalian cells. The importance of primary cilium to the pathogenesis of PKD was brought to light by studies that demonstrated that loss or mutations in genes affecting cilia structure, composition, and function produce cysts in mammalian kidney tubules [17,18]. Subsequently, PC1 and PC2 proteins were identified at the primary cilium, where they play a vital role in signaling [19,20]. There exists a complex relationship between primary cilia and cyst growth in PKD. While some studies report that ablation or dysfunctional cilia alone induces kidney cysts [18,21,22], others show that ablation of both primary cilia and polycystins delay cyst growth in ADPKD mouse models [23,24]. Though an established relationship exists between primary cilia and cyst growth, how primary cilia are regulated in ADPKD remain unclear.

In addition to centrosome aberrations, alterations in epigenetic regulation have also been suggested to play a significant role in the progression of cyst growth in ADPKD. These mechanisms act to modify the chromatin state and accessibility of DNA to transcription factors. Evidence for alterations in the epigenetic control of gene expression in ADPKD is accumulating, and emerging data, particularly in the area of histone modification (such as acetylation and methylation of histone proteins), support the importance of abnormal epigenetic regulation in ADPKD [23]. This is evidenced by the participation of epigenetic modulators in the regulation of transcriptional and/or post-translational activities of PKD-associated signaling pathways, and the determination that inhibition of these epigenetic modulators delays cyst growth in PKD mouse models [25,26]. Although these studies have been critical in providing mechanistic insights, and identifying potential epigenetic targets in ADPKD, relatively few studies have explored the role of epigenetic modulation in centrosome integrity. Therefore, identifying epigenetic factors and establishing how they regulate genome instability is significant for our understanding of disease heterogeneity and cyst progression, and for the identification of therapeutic targets for ADPKD.

The SET and MYND-domain (SMYD) lysine methyltransferase family is a subgroup of histone lysine methyltransferases (KMTs) that play important roles in gene expression and protein function by acting as transcription factors and targeting histone and non-histone molecules for methylation [27]. The SMYD family consists of five members that are known to play a critical role in a variety of tumors [28]. The SMYD3 protein is frequently overexpressed in human cancers and has been reported to drive cell cycle progression and cell proliferation [29,30], and more recently, maintain genome integrity [31]. The molecular mechanisms by which SMYD3 exerts its functions are attributed to epigenetic control of gene expression through the methylation of histones (H3K4, H4K5, and H4K20) [32,33,34], and the methylation of non-histone proteins, such as MAP3K2 (mitogen-activated protein kinase 2) [35] and VEGFR1 (vascular endothelial growth factor receptor 1) [36] in cancer development. Inhibition of SMYD3 has therapeutic potential for cancer treatment [30]. However, whether SMYD3 plays a role in the regulation of ADPKD pathology remains unknown.

This study was designed to gain insights into the role of SMYD3 in ADPKD. We identified the upregulation of SMYD3 as an important contributor to cell proliferation and cystogenesis and determined a regulatory mechanism by which SMYD3 modulates genome instability in ADPKD. We propose that pharmacological inhibition of SMYD3 represents a promising strategy for the treatment of ADPKD.

## 2. Materials and Methods

### 2.1. Cell Culture

Mouse inner medullary collecting duct (mIMCD3), NIH3T3 fibroblasts, and human embryonic kidney 293 (HEK293T) cells were maintained at 37 °C in 5% CO_2_ in DMEM (Invitrogen, Waltham, MA, USA) supplemented with 10% FBS. *PKD1* heterozygous PH2 and *PKD1* homozygous PN24 cells (provided by S. Somlo through the George M. O’Brien Kidney Center, Yale University, New Haven, CT, USA) were cultured as described previously [37]. For immunofluorescence analysis, cells were plated at 30% confluence in plates containing glass coverslips, grown until 60% confluent, and either stained or transfected with GFP-tagged plasmid for 24–48 h, followed by immunostaining.

### 2.2. Plasmids

The GFP-tagged WT α-tubulin plasmid was purchased from Addgene, Watertown, MA, USA. The GFP-tagged SMYD3 and Flag-tagged SMYD3 plasmids were constructed by cloning full-length SMYD3 into the pAcGFP-C1 vector (Clontech Laboratories, Inc. Mountain View, CA, USA) and pCMV-C-Flag vector.

### 2.3. Generating a Stable SMYD3-Knockdown IMCD3 Cell Line

Lentiviral plasmid pGIPZ-shSMYD3 carrying SMYD3 shRNA, or the control empty vector pGIPZ-NS, were co-transfected with psPAX2 packaging plasmid, and pMD2.G envelope plasmid, in HEK293T cells using calcium phosphate. After 12–16 h, the transfection medium was replaced with normal growth medium (DMEM plus 10% FBS and penicillin/streptomycin) and incubated for 48 h, after which culture medium containing lentiviral particles was collected from the HEK293T cells and centrifuged at 1600× *g* for 10 min. IMCD3 cells were then infected with supernatant containing lentiviral particles together with 5 µg/mL polybrene (Sigma, St. Louis, MO, USA). Twenty-four hours later, the virus-containing medium was replaced with normal growth medium plus 10 µg/mL puromycin. After two days of selection, all of the cells were GFP positive, which indicated high transduction efficiency. Subsequently, the cells were cultured in medium with 10 µg/mL puromycin and used for Western blot and immunostaining analyses.

### 2.4. Antibodies and Reagents

The primary antibodies used in this study included the following: mouse monoclonal antibodies against SMYD3 (Sigma, SAB4200344, 1:300 used for immunofluorescence (IF) and 1:1000 used for Western blot (WB)), acetylated α-tubulin (6-11B-1, Sigma, T7451, 1:4000 used for IF), γ-tubulin (GTU-88, Sigma, T5326, 1:1000 used for IF), α-tubulin (DM1A, sc-32293, 1:1000 used for WB and 1:2000 used for IF), β-tubulin (D-10, sc-5274, 1:1000 used for WB), actin (AC-15, Sigma, A1978, 1:1000), Flag (clone M2, Sigma, F1804, 1:1000 for WB), GFP-tagged (B-2, sc-9996, 1:1000 for WB), CDK4 (3F121, sc-70831, 1:1000 for WB), CDK6 (F-7, sc-390493, 1:1000 for WB), Cyclin D1 (72-13G, sc-450, 1:500 for WB), ERK (CST, no. 4696, 1:1000 used for WB), rabbit polyclonal antibodies against SMYD3 (ThermoFisher Scientific, Rockford, IL, USA, PA5-31919, 1:300 used for IF and 1:1000 used for WB), STAT3 (Santa Cruz Biotechnology Inc., Dallas, TX, USA, sc-482, 1:1000 used for WB), phosphorylated antibodies for ERK-T202/Y204 (CST, Danvers, MA, USA, no. 9101, 1:1000 used for WB), STAT3-Y705 (CST, no. 9131, 1:1000 used for WB), p65-S536 (CST, no. 3031, 1:500 used for WB), and pericentrin (EMD Millipore, St. Louis, MO, USA, ABT59, 1:500 used for IF). Also, we used methylated tubulin antibodies (α-TubK40me3 and α-TubK394me3) generated by our lab (Covance, Princeton, NJ, USA, now Labcorp drug development) and used α-TubK40me3 (1:500 for IF and 1:1000 for WB) and α-TubK394me3 (1:1000 for WB). All secondary antibodies for immunofluorescence (donkey anti-mouse or donkey anti-rabbit, conjugated with Alex Fluor 488 or 555) were purchased from Invitrogen; the secondary antibodies for the Western blot tests, including donkey anti-rabbit IgG–horseradish peroxidase (sc-2313), and goat anti-mouse IgG–horseradish peroxidase (sc-2005), were purchased from Santa Cruz Biotechnology Inc.

### 2.5. Histology and Immunohistochemistry

Paraffin-embedded sections (5 µm) were subjected to H&E staining and immunohistochemistry. For SMYD3 staining, a polyclonal rabbit anti-SMYD3 antibody (ThermoFisher, PA5-31919, 1:100), biotinylated secondary antibody (Santa Cruz Biotechnology Inc., 1:500), and DAB substrate system were used. Kidney sections were counterstained with hematoxylin. Imaging was acquired using a Nikon SMZ800 microscope configured with a 0.5× Achro objective (Nikon Instruments Inc, Melville, NY, USA).

### 2.6. Cell Immunofluorescence Staining

Cells grown on coverslips were rinsed with 1× phosphate-buffered saline (PBS), and either fixed in cold methanol for 10 min at −20 °C, followed by permeabilization with 0.2% Triton X-100 (Sigma) for 15 min at 37 °C, or with 4% paraformaldehyde (PFA) for 10 min at 37 °C, followed by permeabilization with 0.2% Triton X-100 for 10 min at room temperature. Cells were subsequently washed three times with PBS, then blocked in 2% BSA and sequentially incubated with primary and secondary antibodies.

### 2.7. Western Blot Analysis and Immunoprecipitation

Protein extraction for Western blot and immunoprecipitation analyses was performed as previously described [38]. For the immunoprecipitation analysis, anti-SMYD3, anti-GFP-tagged, or anti-Flag-tagged antibodies and their isotype control antibodies were coupled to protein A agarose beads (Pierce, Waltham, MA, USA) in PBS containing 5 mg/mL BSA (Sigma-Aldrich, St. Louis, MO, USA) and processed as previously described [38].

### 2.8. Glutathione S-Transferase (GST) Pull-Down Assay

pGEX-6P-1 constructs were transformed into BL21 (DE3) (Novagen, Burlington, MA, USA). Proteins were expressed and purified using glutathione Sepharose. Purified GST-SMYD3 or its truncations immobilized on glutathione Sepharose beads were incubated with whole-cell lysate in cell lysis buffer (see [38] for recipe) overnight at 4 °C. The next day, the beads were washed with cell lysis buffer, and the immune complexes were eluted from the beads using loading buffer by boiling for 7 min and then subjected to Western blot analysis.

### 2.9. Microscopy and Imaging

Immunofluorescent images were acquired using either an imaging microscope (Nikon TE 2000-U) with a Plan Apochromat 60× 1.49 oil objective (Nikon) or an Elyra PS.1 Super Resolution microscope equipped with a Plan Apochromat 63× 1.49 oil objective (Zeiss, White Plains, NY, USA).

### 2.10. Real-Time Quantitative Reverse Transcription PCR (qRT-PCR)

Total RNA extraction was performed using the RNeasy Plus Mini Kit (QIAGEN, Germantown, MD, USA). cDNA was synthesized using an iScript cDNA Synthesis Kit (Bio-Rad, Hercules, CA, USA) as per the manufacturer’s specifications. RNA expression profiles were analyzed via real-time PCR using iTaq SYBR Green Supermix with ROX (Bio-Rad) in an iCycler iQ Real-Time PCR Detection System, as previously described [38,39]. Samples were run in triplicates per experiment, and each experiment was repeated 3 times. Target gene levels were normalized to those of actin.

### 2.11. RNA Interference

RNA oligonucleotides that specifically targeted mouse SMYD3 were purchased from Santa Cruz Biotechnology Inc. The RNA oligonucleotides were transfected using Lipofectamine RNAiMAX (Invitrogen) following the manufacturer’s instructions. Then, 48 h after transfection, cells were harvested and analyzed via Western blotting.

### 2.12. Mice

*Pkd1^fl/fl^* mice and *Ksp-Cre* transgenic mice were generated as described previously [40]. *Pkd1^fl/fl^*:*Ksp-Cre* mice were generated as previously described [26]. *Smyd3^fl/fl^* mice were generated with sperm ordered from the Knockout Mouse Project (KOMP), Jackson Laboratory. The *Smyd3^fl/fl^* mice developed normally in size and behavior and were fertile. *Pkd1^fl/fl^:Smyd3^fl/fl^:Ksp-Cre* mice were generated by first crossing the *Pkd1^fl/+^:Ksp-Cre* mice with the *Smyd3^fl/fl^* mice to obtain *Pkd1^fl/+^:Smyd3^fl/+^:Ksp-Cre* mice, which we then back-crossed to obtain the desired genotype.

### 2.13. Study Approval

All animal protocols were approved by and conducted in accordance with Laboratory Animal Resources of Mayo Clinic and Institutional Animal Care and Use Committee regulations (protocol A00003756-18-R21).

### 2.14. Measurement of Cyst Area

The cyst area was quantified in whole kidneys after H&E staining using ImageJ NIH software (NIH, USA, http://imagej.org, (accessed on 3 March 2024)). The cyst area was calculated using the following formula: (cyst area/total area) × 100%. Three sections from both kidneys were analyzed for each mouse.

### 2.15. Quantitative BUN Determination

A QuantiChrom^TM^ Urea Assay Kit (BioAssay Systems, Hayward, CA, USA) was used. Serum samples were diluted 5-fold in distilled water prior to each assay. Next, 5 µL water (blank), 5 µL standard (50 mg/dL), and 5 µL samples were transferred in triplicate into a 96-well plate with a clear bottom. Then, 200 µL of working reagent (prepared in a 1:1 ratio of reagents A and B) was added and tapped lightly to mix. Samples were incubated for 20 min at room temperature and the optical density was measured at a wavelength of 520 nm. Urea and BUN were calculated as follows:

Urea (mg/dL) = [(OD_Sample_ − OD_Blank_)/(OD_Standard_ − OD_Blank_)] × n × [STD]

BUN (mg/dL) = [Urea]/2.14.

where OD_Sample_, OD_Blank_, and OD_Standard_ are the optical density values of the sample, blank and standard, respectively; n is the dilution factor; [STD] = urea standard concentration (mg/dL).

### 2.16. Statistics

All data are presented as their mean ± SD. All statistical analyses were performed using SPSS Statistics 22 software and GraphPad Prism 9.1.0. *p* values were calculated using a 2-tailed unpaired Student’s *t*-test and a 1-way ANOVA; a *p* value less than 0.05 was considered significant.

## 3. Results

### 3.1. SMYD3 Is Upregulated in PKD1 Mutant Renal Epithelial Cells and ADPKD Tissues

**Figure 1:**
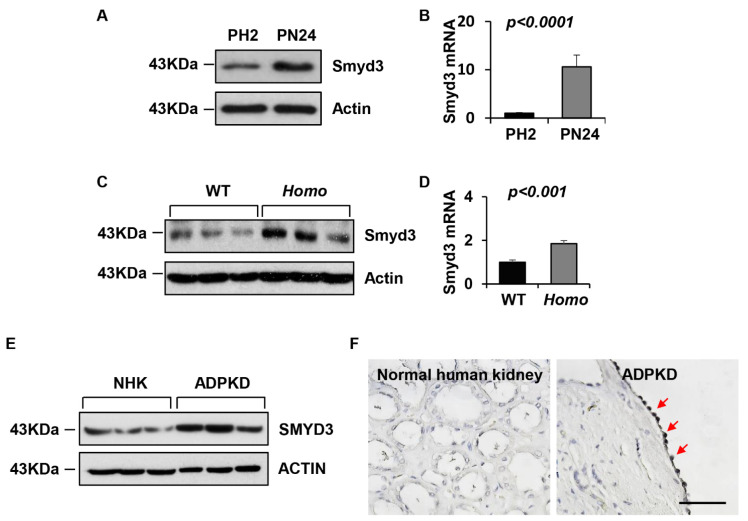
SMYD3 is upregulated in *PKD1* mutant renal epithelial cells and tissues. (**A**) SMYD3 protein and (**B**) mRNA levels in *PKD1* heterozygous (PH2) cells and *PKD1* homozygous null (PN24) cells as analyzed through Western blot and qRT-PCR, respectively. (**C**) SMYD3 protein and (**D**) mRNA levels in postnatal day 7 (P7) kidneys from *Pkd1^+/+^:Ksp-Cre* (WT) and *Pkd1^fl/fl^:Ksp-Cre* (Homo) neonates as analyzed through Western blot and qRT-PCR, respectively. *n* = 3. (**E**) Western blot analysis of SMYD3 levels in kidneys from ADPKD patients and normal human kidney (NHK) tissues. (**F**) Immunohistochemistry analysis indicated that SMYD3 is upregulated in the cyst-lining epithelial cells of human ADPKD kidneys (red arrows). Scale bar: 100 μm.

To evaluate whether SMYD3 plays a role in ADPKD, we first examined its expression in *PKD1* mouse cell lines and kidney tissues, and in human ADPKD kidneys. We found that SMYD3 was upregulated in PN24 (*PKD1* homozygous) cells compared with PH2 (*PKD1* heterozygous) control cells when examined through Western blot and quantitative RT-PCR (qRT-PCR) analyses (Figure 1A,B). SMYD3 protein and mRNA levels were also upregulated in the kidneys from the *Pkd1^fl/fl^:Ksp-Cre* mice, an animal model for ADPKD, compared with age-matched *PKD1* wild-type (WT) kidneys (Figure 1C,D). In addition, SMYD3 was upregulated in kidneys from ADPKD patients compared with normal human kidneys (NHKs) (Figure 1E). We further found that SMYD3 was increased in the cyst-lining epithelial cells in kidneys from the ADPKD patients when examined with immunohistochemistry analysis (Figure 1F). These results demonstrate that the expression of SMYD3 is upregulated in renal cystic epithelial cells and tissues, and in cyst linings in ADPKD kidneys.

### 3.2. SMYD3 and PKD1 Double Conditional Knockout Delayed Renal Cyst Growth

**Figure 2:**
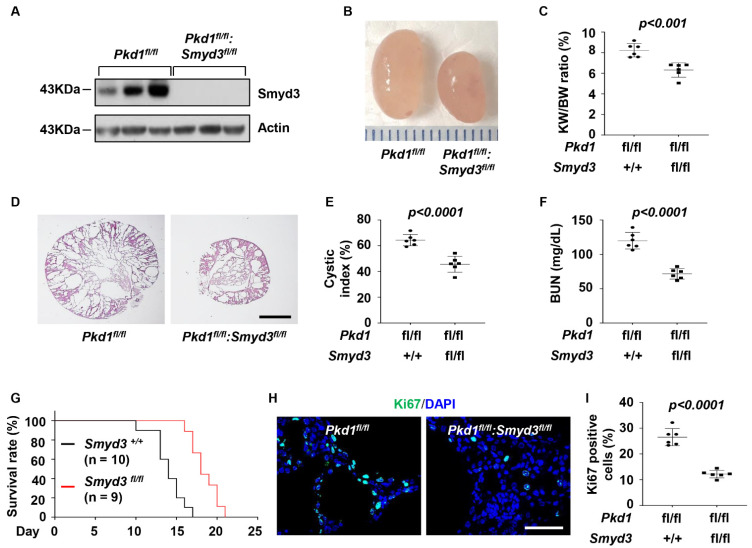
Double conditional knockout of *SMYD3* and *PKD1* delayed renal cyst formation. (**A**) Western blot analysis of SMYD3 expression in kidneys from P7 *Pkd1 ^fl/fl^:Smyd3^+/+^:Ksp-Cre* (*Pkd1^fl/fl^*) and *Pkd1^fl/fl^:Smyd3 ^fl/fl^:Ksp-Cre* (*Pkd1 ^fl/fl^:Smyd3 ^fl/fl^*) mice. (**B**) Representative kidneys from *Pkd1^fl/fl^:Smyd3^+/+^:Ksp-Cre* (*Pkd1^fl/fl^*) and *Pkd1 ^fl/fl^:Smyd3 ^fl/fl^:Ksp-Cre* (*Pkd1^fl/fl^:Smyd3 ^fl/fl^*) mice. Ruler tick marks are in inches. (**C**) KW/BW ratio in P7 *Pkd1 ^fl/fl^:Smyd3 ^fl/fl^:Ksp-Cre* compared to *Pkd1 ^fl/fl^:Smyd3^+/+^:Ksp-Cre* mice. (**D**) Histological examination of kidneys from *Pkd1 ^fl/fl^:Smyd3^+/+^:Ksp-Cre* (*Pkd1^fl/fl^*) and *Pkd1^fl/fl^:Smyd3 ^fl/fl^:Ksp-Cre* (*Pkd1^fl/fl^*:*Smyd3 ^fl/fl^*) mice. Scale bar: 1000 µm. (**E**) Percent cystic area relative to total kidney section area in kidneys from P7 *Pkd1 ^fl/fl^:Smyd3 ^fl/fl^:Ksp-Cre* compared to *Pkd1 ^fl/fl^:Smyd3^+/+^:Ksp-Cre* neonates. (**F**) BUN levels in serum from P7 *Pkd1 ^fl/fl^:Smyd3 ^fl/fl^:Ksp-Cre* compared with *Pkd1 ^fl/fl^:Smyd3^+/+^:Ksp-Cre* mice. (**G**) *Pkd1^fl/fl^:Smyd3^fl/fl^:Ksp-Cre* mice (*Smyd3^fl/fl^*) lived to a mean age of 19 days (*n* = 9), whereas *Pkd1 ^fl/fl^:Smyd3^+/+^:Ksp-Cre* mice (*Smyd3^+/l+^*) died of PKD at a mean age of 15 days (*n* = 10), *p* < 0.01. (**H**) Ki67 staining for cell proliferation in kidneys from P7 *Pkd1 ^fl/fl^:Smyd3^fl/fl^:Ksp-Cre* compared to *Pkd1^fl/fl^:Smyd3^+/+^:Ksp-Cre* mice. (**I**) The percentage of Ki67-positive nuclei in cyst-lining epithelial cells calculated from an average of 500 nuclei per mouse kidney section. Scale bar: 10 μm.

To investigate the functional role of SMYD3 in vivo, we generated *PKD1* and *SMYD3* double conditional knockout *Pkd1^fl/fl^:Smyd3^fl/fl^:Ksp-Cre* (DKO) mice, which had kidney-specific cadherin-driving Cre expression [40]. Deletion of SMYD3 was confirmed in the kidneys from these DKO mice via Western blot analysis (Figure 2A). The knockout of *SMYD3* delayed cyst growth and improved kidney function in postnatal day 7 (P7) DKO kidneys compared to age-matched *PKD1* single knockout (*PKD1* SKO) kidneys, as shown by the decrease in the kidney-weight-to-body-weight (KW/BW) ratio (Figure 2B,C), cystic index (Figure 2D,E), and blood urea nitrogen (BUN) levels (Figure 2F). The DKO mice lived to a mean age of 18 days, while *PKD1* SKO mice died at a mean age of 14 days (Figure 2G). Knockout of SMYD3 decreased the number of Ki67-positive cells in kidneys from the *PKD1* knockout mice (Figure 2H,I). The *SMYD3* SKO mice, however, displayed no phenotypic alterations compared to WT mice at P7 (Figure S1A–D). These results suggest that SMYD3 delays cyst growth and decreases cystic cell proliferation in *PKD1* mutant mouse kidneys.

### 3.3. SMYD3 Regulates PKD-Associated Signaling Pathways in PKD1 Mutant Mouse Kidneys

**Figure 3:**
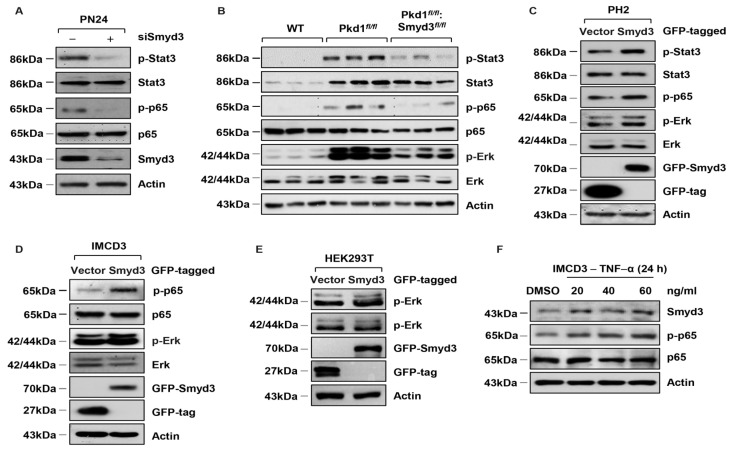
SMYD3 regulates the activation of PKD-associated signaling pathways in *PKD1* mutant renal epithelial cells and tissues. (**A**) Knockdown of SMYD3 with siRNA decreased the phosphorylation of Stat3 and p65 but did not affect their total expression in PN24 cells. (**B**) Western blot analysis of the expression of phosphorylated Erk (p-Erk), phosphorylated Stat3 (p-Stat3), and phosphorylated NF-κB (p-p65) in kidneys from *Pkd1^+/+^:Smyd3^+/+^:Ksp-Cre* (WT), *Pkd1^fl/fl^:Smyd3^+/+^:Ksp-Cre* (*Pkd1^fl/fl^*), and *Pkd1^fl/fl^:Smyd3^fl/fl^:Ksp-Cre* (*Pkd1^fl/fl^:Smyd3^fl/fl^*) neonates at P7. The expression levels of p-Erk, p-Stat3, and p-p65 were increased in kidneys from *Pkd1^fl/fl^:Smyd3^+/+^:Ksp-Cre* mice compared with those from *Pkd1^+/+^:Smyd3^+/+^:Ksp-Cre* mice, whereas the expression of these proteins was decreased in kidneys from *Pkd1^fl/fl^:Smyd3^fl/fl^:Ksp-Cre* compared with *Pkd1^fl/fl^:Smyd3^+/+^:Ksp-Cre* neonates. (**C**–**E**) Overexpression of GFP-tagged SMYD3 increased the expression of p-Stat3, p-p65, and p-ErK in PH2 cells (**C**), increased the expression of p-p65 and p-ErK in mouse IMCD3 cells (**D**), and increased the expression of p-ErK in HEK293T cells (**E**), compared to GFP-vector transfected control cells. (**F**) Stimulation with cytokine TNF-α induced SMYD3 expression in a concentration-dependent manner in mIMCD3 cells.

Next, we investigated whether SMYD3 regulates cell proliferation via PKD-associated signaling pathways. We found that the knockdown of SMYD3 with siRNA decreased the levels of the phosphorylated Stat3 (p-Stat3) and p65 subunit of NF-κB (p-p65) but not their total (Stat3 and p65) in *PKD1* mutant cells (Figure 3A). We also found that p-Stat3, p-p65, and phosphorylated Erk1/2 (p-Erk1/2) were increased in *PKD1* SKO tissues (Figure 3B), and DKO decreased the levels of p-Stat3, p-p65, and p-Erk1/2, but had no effect on the total levels of Stat3, p65, and Erk1/2 in *PKD1* mutant kidney tissues (Figure 3B). We also found that overexpression of GFP-tagged SMYD3 (GFP-SMYD3) increased the phosphorylation of Stat3, p65, and Erk1/2 in PH2 cells (Figure 3C) and also increased the phosphorylation of p65 and Erk1/2 mIMCD3 cells (Figure 3D). In addition, overexpression of GFP-SMYD3 increased the phosphorylation of Erk1/2 in HEK293T cells (Figure 3E). NF-κB, through the regulation of downstream cytokines, is known to affect signal transduction. Tumor necrosis factor-α (TNF-α) is particularly important because it stimulates cyst growth in vivo, and it forms a positive feedback loop with NF-κB. We found that treatment with TNF-α increased the expression of SMYD3 in mIMCD3 cells (Figure 3F). Together, our results suggest that SMYD3 contributes to the activation of Stat3, NF-κB, and Erk1/2 signaling pathways in *PKD1* mutant kidneys, and NF-κB mediates the regulation of SMYD3 expression.

### 3.4. SMYD3 Regulates Cell Cycle Effectors

**Figure 4:**
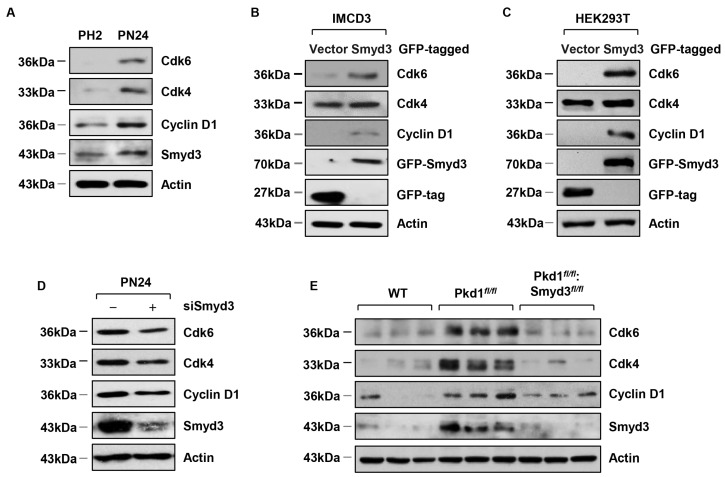
SMYD3 regulates the expression of cell cycle effectors in *PKD1* mutant renal epithelial cells and tissues. (**A**) Western blot analysis of the expression of the cyclin-dependent kinases Cdk4 and Cdk6 and Cyclin D1 in PH2 cells and PN24 cells. Cdk4, Cdk6, and Cyclin D1 were increased in PN24 cells. (**B**,**C**) Overexpression of GFP-tagged SMYD3 increased the expression of Cdk4, Cdk6, and Cyclin D1 in mIMCD3 cells (**B**), and in HEK293T cells (**C**). (**D**) Knockdown of SMYD3 with siRNA decreased the expression of Cdk4, Cdk6, and Cyclin D1 in PN24 cells. (**E**) Western blot analysis of the expression of Cdk4, Cdk6, and Cyclin D1 in kidneys from *Pkd1^+/+^:Smyd3^+/+^:Ksp-Cre* (WT), *Pkd1^fl/fl^:Smyd3^+/+^:Ksp-Cre* (*Pkd1^fl/fl^*), and *Pkd1^fl/fl^:Smyd3^fl/fl^:Ksp-Cre* (*Pkd1^fl/fl^:Smyd3^fl/fl^*) neonates at P7.

SMYD3 has been reported to target proteins involved in the cell cycle to subsequently regulate cell proliferation. We therefore hypothesized that SMYD3 could also regulate the levels of cell cycle proteins in PKD. Before characterizing the role of SMYD3 in cell cycle proteins, we first evaluated the protein levels of cyclin-dependent kinases (Cdks), primary regulators of the cell cycle, in *PKD1* mutant renal epithelial cells. We found that Cdk6, Cdk4, phosphorylated Cdk2, and Cyclin D1 were all upregulated in PN24 cells compared to PH2 cells (Figure 4A). We further found that overexpression of GFP-SMYD3 resulted in increased levels of Cdk6, Cdk4, phosphorylated Cdk2, and Cyclin D1 (Figure 4B) in mIMCD3 cells, and in HEK293T cells (Figure 4C). Next, we evaluated the effect of SMYD3 on these cell cycle effectors in *PKD* mutants. We found that knockdown of SMYD3 in PN24 cells resulted in the downregulation of Cdk6, Cdk4, phosphorylated Cdk2, and Cyclin D1 (Figure 4D). In addition, compared to wild-type kidneys, we found that Cdk6, Cdk4, phosphorylated Cdk2, and Cyclin D1 were upregulated in *PKD1* SKO kidneys, and DKO resulted in their downregulation (Figure 4E). These results suggest that SMYD3 may contribute to cell cycle regulation in *PKD1* mutant mouse kidneys.

### 3.5. SMYD3 Is Located at the Centrosome and Regulates Centrosome Amplification

**Figure 5:**
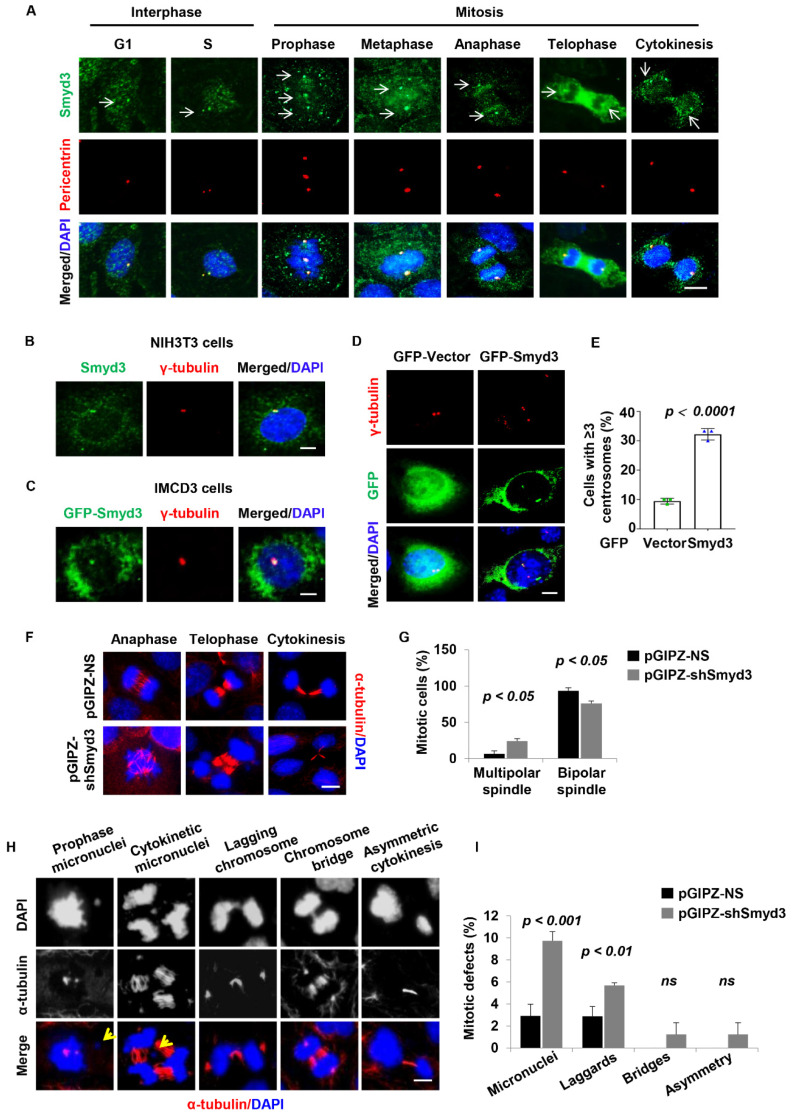
SMYD3 regulates centrosome biogenesis and mitotic fidelity. (**A**) Representative images of IMCD3 cells co-stained with SMYD3 (green—indicated by white arrows) and centrosome marker pericentrin (red) in G1, S, and mitotic phases. Scale bar: 10 μm. (**B**) Representative images of NIH3T3 fibroblast cells co-stained with SMYD3 (green), or co-stained with centrosome marker γ-tubulin (red). Scale bar: 10 μm. (**C**) Representative images of IMCD3 cells showing expression of exogenous GFP-SMYD3 (green), co-stained with centrosome marker γ-tubulin (red). Scale bar: 10 μm. (**D**,**E**) Representative images (**D**) and quantitative analysis (**E**) of IMCD3 cells expressing GFP-SMYD3 (green), co-stained with centrosome marker γ-tubulin (red). Overexpression of SMYD3 resulted in centrosome amplification (cells with ≥3 centrosomes) (*n* = 50) in GFP-SMYD3-overexpressed IMCD3 cells compared to GFP-vector control cells. Scale bar: 10 μm. (**F**,**G**) Knockdown of SMYD3 resulted in aberrant mitotic spindle formation. Representative images (**F**) and quantitative analysis (**G**) of the percentage of cells with multipolar spindles in SMYD3-knockdown IMCD3 cells compared to control cells. Scale bar: 20 μm. (**H**,**I**) Knockdown of SMYD3 resulted in defective mitosis/cytokinesis in IMCD3 cells stained with α-tubulin (red) and DAPI (blue). Representative images (**H**) and quantification (**I**) of the percentage of cells with mitotic/cytokinesis abnormalities, including chromosome bridges, micronuclei, and laggards, in SMYD-knockdown IMCD3 cells compared to control cells. Yellow arrows indicate micronuclei; ns represents not significant. Scale bar: 20 μm.

Cell cycle-dependent kinases such as Cdk2 and Cdk4 have been reported to enhance centrosome duplication and amplification [41]. Since SMYD3 modulated the expression of Cdk4, we hypothesized that SMYD3 could play a role in centrosome biogenesis. We found that SMYD3 was enriched at the centrosome in interphase cells, and at the spindle poles in the G1, S, and mitotic/cytokinetic phases of the cell cycle in mIMCD3 cells (Figure 5A). The centrosome localization was confirmed in mouse fibroblast NIH3T3 cells with a second SMYD3 antibody (Figure 5B). To rule out non-specificity of the antibodies, we exogenously expressed GFP-SMYD3 in mIMCD3 cells and found that it also localized to the centrosome (Figure 5C). Interestingly, we found that overexpression of SMYD3 resulted in aberrant centrosome amplification in mIMCD3 cells (Figure 5D,E).

### 3.6. SMYD3 Is Required for Normal Mitosis and Cytokinesis

The presence of extra centrosomes is reported to result in mitosis and cytokinesis defects. We therefore hypothesized that SMYD3 could modulate mitosis and cytokinesis. To assess this, we stably knocked down SMYD3 with shRNA in mIMCD3 cells. The efficiency of SMYD3 silencing was confirmed with qRT-PCR and Western blot tests (Figure S2A), and immunostaining analysis (Figure S2B). We found that the depletion of SMYD3 resulted in a significant increase in mitotic and cytokinesis defects in the mIMCD3 cells. These defects included an increased formation of multipolar spindles (Figure 5F,G), increased formation of micronuclei in cytokinesis, the failure of chromosomes to congress at the prophase, and the lagging of chromosomes (Figure 5H,I). These results suggest that depletion of SMYD3 plays a role in mitotic spindle organization and the ensuing mitosis and cytokinesis. The accumulation of mitosis/cytokinesis defects is known to result in genome instability, which may subsequently lead to apoptotic cell death. We therefore evaluated whether knockout of SMYD3 induced cystic epithelial cell death. The knockout of *SMYD3* in *PKD1* mutant mouse kidneys induced cyst-lining epithelial cell apoptosis as analyzed by TUNEL (terminal deoxynucleotidyl transferase dUTP nick end-labeling) staining (Figure S2C,D). These results demonstrate that depletion of SMYD3 increases mitosis and cytokinesis defects and causes cell death.

### 3.7. SMYD3 Interacts with Methylates α-Tubulin at Lysine 40

**Figure 6:**
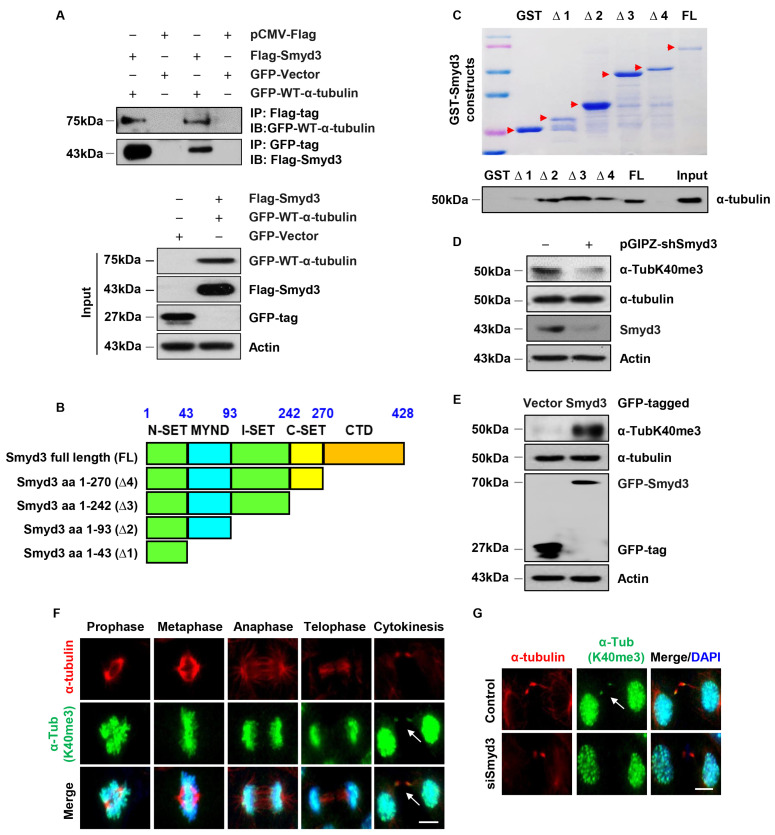
SMYD3 interacts with and regulates the methylation of α-tubulin. (**A**) Co-immunoprecipitation analysis revealed the interaction between Flag-SMYD3 and GFP-WT-α-tubulin in HEK293T cells. (**B**) Schematic of GST-SMYD3 fusion protein constructs. (**C**) The expression of GST-SMYD3 fusion protein was detected with Coomassie blue staining (top panel, red arrows indicate expression of truncated proteins), and GST pull-down assays of GST-SMYD3 fusion proteins incubated with 1 mg cell lysate from RCTE cells and immunoblotted using α-tubulin antibody (bottom panel). (**D**) Knockdown of SMYD3 decreased the methylation of α-tubulin at K40 in IMCD3 cells compared to control cells when examined using Western blot analysis. (**E**) Overexpression of GFP-tagged SMYD3 increased the level of methylation of α-tubulin at K40 in IMCD3 cells compared to that in GFP-tagged vector transfected control cells when examined using Western blot analysis. (**F**) Representative images indicating that microtubule methylation occurs during mitosis and cytokinesis in IMCD3 cells co-stained with α-tubulin (red) and α-TubK40me3 (green), and counterstained with DAPI (blue). White arrows indicate endogenous α-tubulin merging with K40 methylated α-tubulin at cytokinesis. Scale bar: 10 μm. (**G**) Representative images indicating that the methylation of mitotic microtubules at cytokinesis was lost in SMYD3 knockdown IMCD3 cells co-stained with α-tubulin and α-TubK40me3. Scale bar: 10 μm.

To investigate how SMYD3 regulates mitotic spindle organization, we evaluated the relationship between SMYD3 and tubulin by first performing co-immunoprecipitation analysis. Since the molecular weight of SMYD3 and the tubulin subunits are close to that of IgG, we used tagged SMYD3 and tubulin plasmids. We found that Flag-tagged SMYD3 (Flag-SMYD3) could pull down GFP-tagged wild-type α-tubulin (GFP-WT-α-tubulin) and GFP-WT-α-tubulin could also pull down Flag-SMYD3 in HEK293T cells (Figure 6A). We also found that endogenous β-tubulin could pull down GFP-SMYD3 in HEK293T cells (Figure S3A), whereas SMYD3 could not pull down β-tubulin. This suggests that SMYD3 forms a direct interaction with α-tubulin and an indirect interaction with β-tubulin owing to its dimerization to α-tubulin. To identify the interaction domain required for the interaction between SMYD3 and α-tubulin, we generated a series of glutathione S-transferase (GST) fusion protein constructs containing mouse SMYD3 fragments (Figure 6A,B, top panel). Our GST pull-down assay indicated that the Myeloid-Nervy-DEAF-1 (MYND) domain (aa43-93) of SMYD3 was required for its binding with α-tubulin (Figure 6C, bottom panel). These results suggest that α-tubulin is a novel substrate for SMYD3.

Next, we tested whether SMYD3 could methylate α-tubulin. To do this, we generated and validated polyclonal antibodies against methylated lysine 40 of α-tubulin (α-TubK40me3) and methylated lysine 394 of α-tubulin (α-TubK394me3), as previously described [38,42]. Depletion of SMYD3 decreased the methylation of α-tubulin at K40 (Figure 6D), and overexpression of GFP-SMYD3 increased the methylation of α-tubulin at K40 (Figure 6E) in mIMCD3 cells. We further showed that the α-TubK40me3 antibody could pull down α-tubulin (Figure S3B) in HEK293T cells. This suggests that SMYD3 methylates α-tubulin at K40.

### 3.8. SMYD3 Is a Mitotic Microtubule Methyltransferase

Methylation of α-tubulin has been reported to modulate microtubule stability and mitosis. We found that mitotic microtubules could be methylated at the K40 site of α-tubulin as stained with our newly generated α-TubK40me3 antibody and co-stained with an α-tubulin antibody. Methylation of mitotic microtubules becomes especially evident during cytokinesis when the spindle midzone compacts into the midbody (Figure 6F, arrows in right panels). In addition, we found that SMYD3 co-localized with α-TubK40me3 at the midbody during cytokinesis (Figure S3C, arrows in bottom panels) in a pattern similar to that observed for α-tubulin and α-TubK40me3 staining. Knockdown of SMYD3 resulted in the loss of the methylation of the mitotic microtubules at the midbody during cytokinesis (Figure 6G). These results suggest that the loss of methylation of α-tubulin at K40 mediated by SMYD3 may contribute to the mitosis and cytokinesis defects observed in SMYD3-knockdown cells.

### 3.9. SMYD3 Regulates Primary Cilium Assembly in PKD1 Mutant Mouse Kidneys

**Figure 7:**
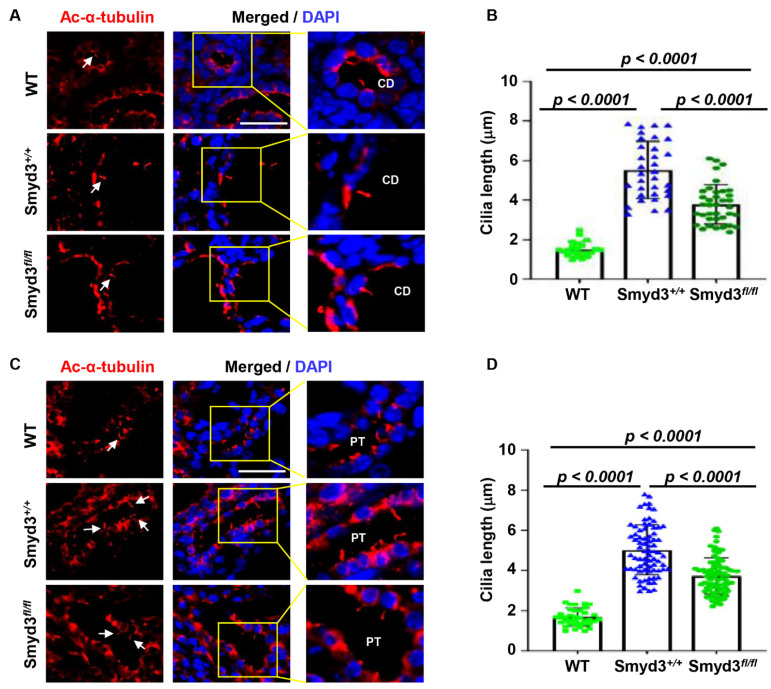
SMYD3 regulates ciliogenesis. (**A**–**D**) SMYD3 regulates ciliogenesis in PKD. Paraffin-embedded kidney sections were immunostained with the ciliary marker acetylated α-tubulin (Ac-α-tub and cilia are indicated by white arrowheads). Representative images (**A**) and quantitative data (**B**) of cilium length in kidney collecting ducts (CDs) in *Pkd1^+/+^:Smyd3^+/+^:Ksp-Cre* (WT, *n* = 25), *Pkd1^fl/fl^:Smyd3^+/+^:Ksp-Cre* (*Smyd3^+/+^*, *n* = 35), and *Pkd1^fl/fl^:Smyd3^fl/fl^:Ksp-Cre* (*Smyd3^fl/fl^*, *n* = 40) kidneys at P7. Representative images (**C**) and quantitative data (**D**) of cilium length in kidney proximal tubules (PT) in *Pkd1^+/+^:Smyd3^+/+^:Ksp-Cre* (WT, *n* = 40), *Pkd1^fl/fl^:Smyd3^+/+^:Ksp-Cre* (*Smyd3^+/+^*, *n* = 75), and *Pkd1^fl/fl^:Smyd3^fl/fl^:Ksp-Cre* (*Smyd3^fl/fl^*, *n* = 75) kidneys at P7. The quantitative data on cilium lengths were measured in five to ten regions for each mouse kidney (*n* = 3). Scale bar: 20 μm.

Dysregulation of cilia biogenesis and function has been proposed as a mechanism for cyst growth in PKD [18]. Since we observed SMYD3 at the centrosome, we investigated whether SMYD3 could play a role in the regulation of ciliogenesis in PKD. To evaluate this, we performed immunostaining to assess the primary cilium phenotype in our *PKD1* SKO and *PKD1:SMYD3* DKO mouse kidneys. We found that knockout of *PKD1* resulted in increased cilia length in the collecting ducts (Figure 7A,B) and in the proximal tubules (Figure 7C,D) of the *Pkd1^fl/fl^:Ksp-Cre* kidneys compared to wild-type kidneys. In addition, knockout of *SMYD3* decreased cilia length in the collecting ducts (Figure 7A,B) and in the proximal tubules of *Pkd1^fl/fl^:Smyd3^fl/fl^:Ksp-Cre* kidneys compared to *PKD1* SKO. Also, we found that the single knockout of *SMYD3* decreased cilia length in the collecting duct (Figure S4A,B) and in the proximal tubules (Figure S4C,D) compared to WT kidneys. These results suggest that SMYD3 contributes to the regulation of ciliogenesis in *PKD1* mutant kidneys.

## 4. Discussion

**Figure 8:**
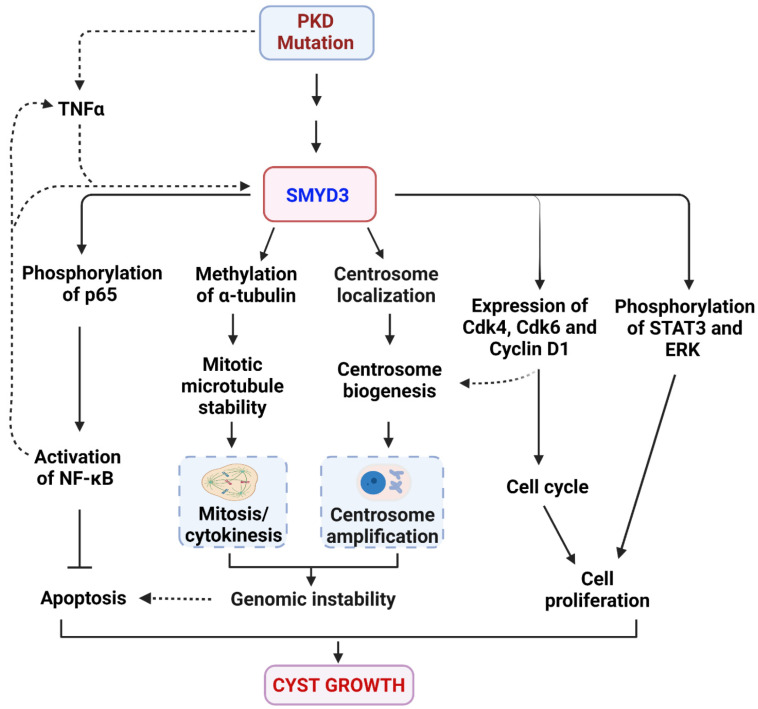
Working model of SMYD3 in the regulation of cyst growth in ADPKD. A schematic representation of the roles and mechanisms of SMYD3 in the regulation of cyst growth in *PKD1* mutant kidneys. *PKD1* knockout or mutation results in the upregulation of SMYD3, which may be induced by TNF-α in cyst fluid. Upregulated SMYD3 in *PKD1* mutant renal epithelial cells (i) regulates the phosphorylation and activation of NF-κB, which represses cystic renal epithelial cell apoptosis; (ii) regulates the phosphorylation and activation of Erk and Stat3 signaling, leading to cystic renal epithelial cell proliferation; and (iii) increases the levels of cyclin-dependent kinases Cdk4 and Cdk6, and Cyclin D1, which modulate the cell cycle, leading to cystic renal epithelial cell proliferation. In addition, (a) SMYD3 is localized at the centrosome, where it modulates centrosome duplication/amplification, and (b) methylates α-tubulin and regulates mitosis/cytokinesis by stabilizing mitotic spindle formation, thereby affecting genomic instability. Together, our results suggest that SMYD3 may promote cyst growth by regulating PKD-associated signaling pathways and chromosomal instability in ADPKD. Created with BioRender.com.

Autosomal dominant polycystic kidney disease (ADPKD) is the most common kidney disease and is characterized by fluid-filled cysts. Worldwide, the frequency of ADPKD is about 1 in 1000 individuals, with approximately half of these individual cases progressing to end-stage renal disease. The main mechanism of ADPKD is the genetic mutation of the *PKD1* and *PKD2* genes. However, the disease course is highly variable, and high chromosomal instability has been suggested to contribute to this variability. Therefore, epigenetic enzymes/mechanisms are proposed to play a key role in regulating disease progression. In this study, we demonstrate that the histone/lysine methyltransferase Smyd3 is an unstudied epigenetic factor that regulates ADPKD pathogenesis. We showed that SMYD3 is upregulated in *PKD1* mutant renal epithelial cells and tissues, correlating with ADPKD disease progression. We have provided evidence that the double conditional knockout of *PKD1* and *SMYD3* delays cyst growth, improves renal function, and normalizes PKD-associated signaling pathways and cell cycle effectors. We further showed that SMYD3 is located at the centrosome and engages in the modulation of genome instability (centrosome amplification and mitosis/cytokinesis defects) and ciliogenesis in cystic kidneys (Figure 8). Our data suggest that pharmacologically inhibiting SMYD3 may be a novel therapeutic strategy for ADPKD treatment.

Our team has previously demonstrated that the lysine methyltransferase SMYD2 regulates the phosphorylation and activation of p65 and Stat3 through methylation of the lysine residues in these proteins [26]. In this study, we found that SMYD3 also regulates the activation of Stat3 and the p65 subunit of NF-κB in *PKD1* mutant renal epithelial cells and tissues, potentially via methylation of these proteins. Interestingly, we found that the treatment of mIMCD3 cells with TNF-α, which has been demonstrated to enhance cyst growth [43], increased the expression of SMYD3, suggesting a positive feedback loop of SMYD3/TNF-α/NF-κB/SMYD3, in a similar manner to SMYD2 [26]. SMYD3 and SMYD2 are the only two members of the SET and MYND-containing protein (SMYD) family to be expressed in the kidney [26], which may explain why they share a functional redundancy in the regulation of PKD-associated signaling pathways, and in their regulatory mechanisms.

Chromosomal instability, driven in part by centrosome amplification, is a reported hallmark of ADPKD and has been hypothesized to result in the generation of clonal heterogeneity, which in turn may facilitate the development of cyst progression [6,14]. In this study, we identified SMYD3 as a centrosomal protein, and its overexpression in mIMCD3 cells resulted in centrosome amplification. These observations implicate SMYD3 localization and expression in maintaining the functional properties and mechanisms associated with centrosome duplication. Deregulation of cell cycle checkpoints has also been reported to cause alterations to the centrosome cycle, resulting in centrosome amplification [44]. Cdk4, for example, along with Cdk2, has been reported to play a role in the regulation of centrosome amplification, where knocking out Cdk4 and Cdk2 was found to restore the normal centrosome phenotype in p53 null cells [41] and breast cancer cells [45]. The correlation between aberrant centrosome amplification and upregulation of cell cycle effectors in SMYD3-overexpressed mIMCD3 cells, and the upregulation of SMYD3 in *PKD1* mutant renal epithelial cells and tissues, suggests that a SMYD3-mediated mechanism may be involved in the centrosome amplification phenotype observed in ADPKD kidneys.

There is evidence to support an essential but complex role of the primary cilia in PKD pathogenesis. Studies have shown that the loss of both primary cilia and either of the polycystins results in a milder cystic phenotype when compared to the individual loss of primary cilia or the polycystins [23]. These studies have led to the proposition that there exists a *PKD1* mutation cilia-dependent cyst activation (CDCA) signaling, required for full activation and cyst growth in *PKD1* knockout mutants [46,47]. Based on this model, it is speculated that the factors that destabilize primary cilia should also reduce cyst growth. In this study, we showed that the average cilia length in *PKD1:SMYD3* double mutants was decreased, correlating with a decreased cystic burden compared to *PKD1* knockout mutants. This suggests that SMYD3 plays a role in the regulation of primary cilia in PKD and may contribute to CDCA signaling.

Previous studies have demonstrated the requirement of methylation in the maintenance of genomic stability and the integrity of the tubulin and histone codes [38,42]. Methylation of α-tubulin at lysine 40 (K40) by the histone lysine methyl transferase SETD2, for example, has been reported to regulate mitosis/cytokinesis [42], and methylation of α-tubulin at lysine 394 (K394) by SMYD2 has been shown to regulate microtubule stability [38]. Here, we show that SMYD3 regulated mitosis/cytokinesis, associated with the methylation of α-tubulin at lysine 40. Our study identifies SMYD3 as a novel mitotic microtubule methyltransferase and suggests that SMYD3 may not be an autonomously acting initiator of centrosome amplification and cytokinesis defects, but rather an essential cooperating partner of other factors that drive these processes, thereby regulating genome instability. With its already-known roles in chromatin remodeling, we propose that SMYD3 is a novel epigenetic factor with a dual function in both chromatin and cytoskeletal remodeling in ADPKD.

In summary, our study demonstrates that SMYD3 is an epigenetic factor that is important for cyst growth in ADPKD. Overexpression of SMYD3 has been associated with poor tumor prognosis and is being pursued as a therapeutic target for cancer. The overexpression of SMYD3 in *PKD* mutant renal epithelial cells and tissues makes it a promising target for the development of a novel ADPKD therapy. Moreover, the involvement of SMYD3 in ciliogenesis broadens our view of its role beyond epigenetic regulation in human diseases.

## Data Availability

The authors declare that all data supporting the findings of this study are available within the article and the Supplementary Data, or from the corresponding author upon reasonable request.

## Supplementary Materials

The following supporting information can be downloaded at: https://www.mdpi.com/article/10.3390/biomedicines12030603/s1. Figure S1: Single conditional knockout of *SMYD3* has no kidney phenotype; Figure S2: Double conditional knockout of *SMYD3* and *PKD1* induced cyst-lining epithelial cell apoptosis; Figure S3: SMYD3 forms a complex with α-tubulin and β-tubulin; Figure S4: Single conditional knockout of *SMYD3* inhibits ciliogenesis.
