# Supplementary material for: Overexpression of SMYD3 Promotes Autosomal Dominant Polycystic Kidney Disease by Mediating Cell Proliferation and Genome Instability
Source: Biomedicines. 2024 Mar 7;12(3):603. doi: 10.3390/biomedicines12030603 (PMC10968571; doi:10.3390/biomedicines12030603)
Supplement: Supplementary file 1 [file biomedicines-12-00603-s001.zip › biomedicines-2895542-supplementary.pdf]

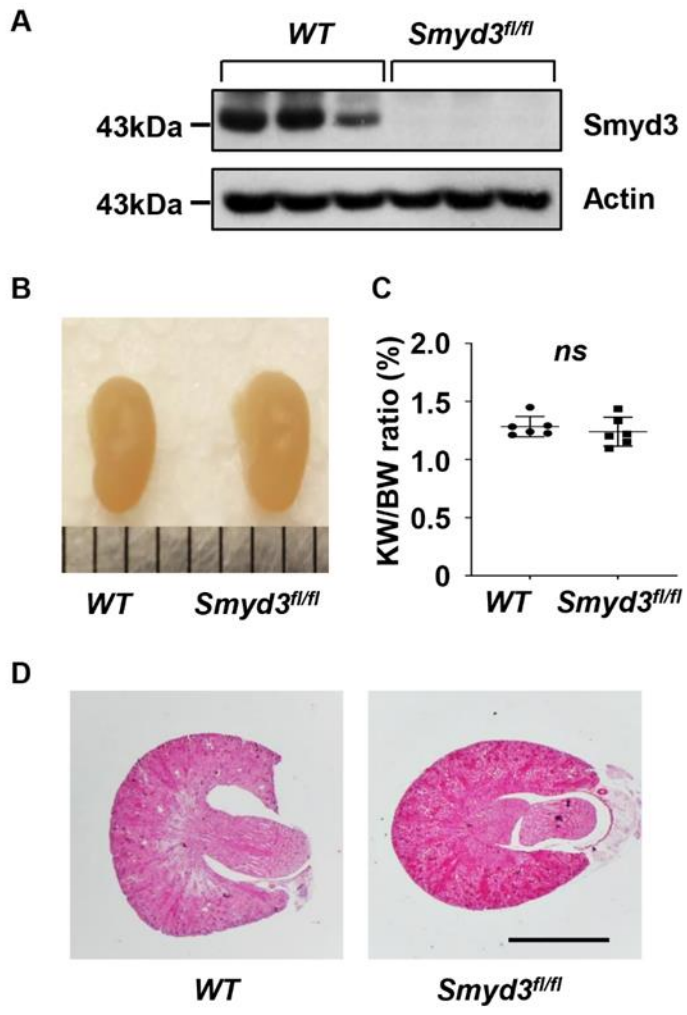

Supplemental Figure S1. Single conditional knockout of *SMYD3* has no kidney phenotype. (**A**) Western blot analysis of SMYD3 expression in P7 kidneys from *Pkd1* $^{+/+}$*:Smyd3*$^{+/+}$*:Ksp-Cre* wild type (*WT)* neonates and *Pkd1*$^{+/+}$*:Smyd3* $^{fl/fl}$*:Ksp-Cre SMYD3* single knockout (*Smyd3* $^{fl/fl}$) neonates. (**B**) Representative kidneys from WT and *SMYD3* single knockout neonates. Ruler tick marks are in inches. (C) KW/BW ratios were not significantly different in P7 *Pkd1*$^{+/+}$*:Smyd3* $^{+/+}$*:Ksp-Cre* versus *Pkd1* $^{+/+}$*:Smyd3* $^{fl/fl}$*:Ksp-Cre* neonates. (D) Representative images for H&E-stained kidney sections from *Pkd1* $^{+/+}$*:Smyd3*$^{+/+}$*:Ksp-Cre and Pkd1* $^{+/+}$*:Smyd3* $^{fl/fl}$*:Ksp-Cre* neonates. Images demonstrate non-cystic kidneys at P7. Scale bar, 1000 μm.

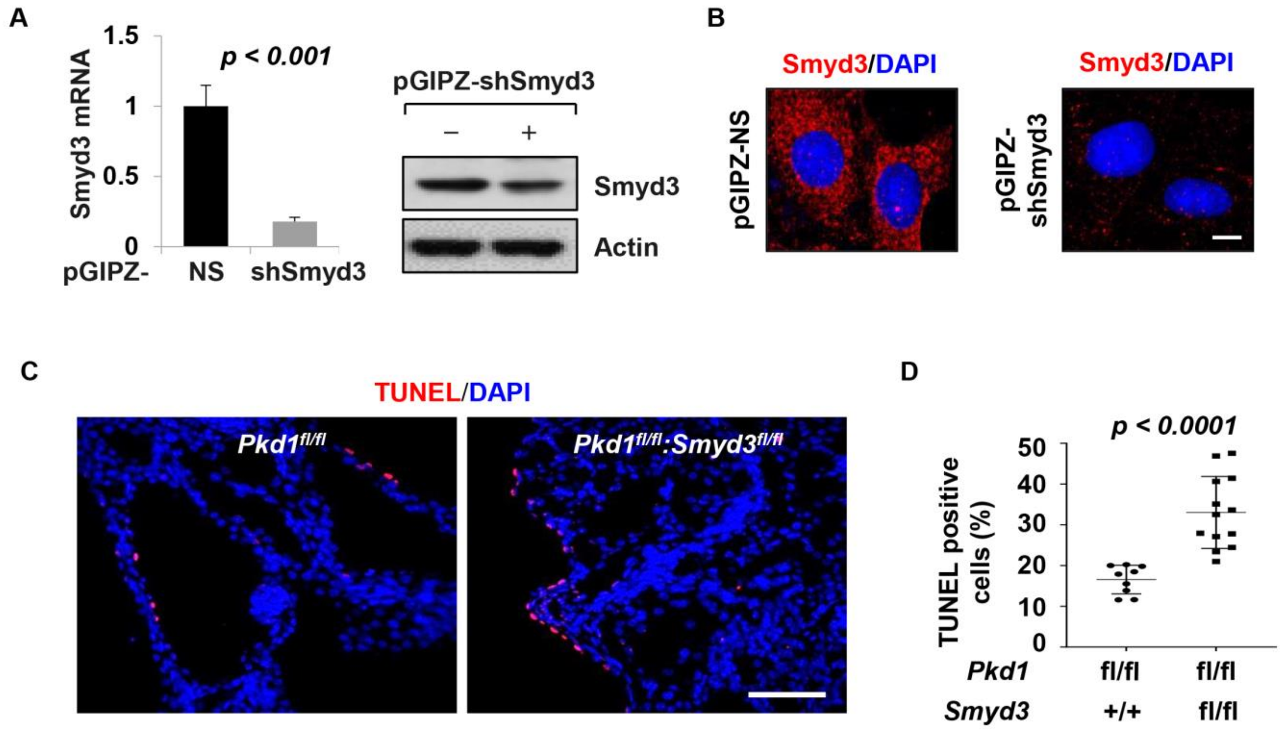

Supplemental Figure S2. Double conditional knockout of *SMYD3* and *PKD1* induced cyst-lining epithelial cell apoptosis. (**A** and **B**) Mouse IMCD3 cells were transduced with Lentivector-mediated SMYD3 shRNA, pGIPZ–shSMYD3, and the control vector, pGIPZ-NS, respectively. The knockdown efficiency of SMYD3 was confirmed by qRT-PCR and western blot (**A**), and by immunostaining (**B**) with SMYD3 antibody (red) and DAPI (blue). Scale bars, 10 μm. (**C** and **D**) Knockout of *SMYD3* induced cyst-lining epithelial cell death in kidneys from P7 *Pkd1$^{fl/fl}$:Smyd3$^{fl/fl}$:Ksp-Cre* neonates. Representative images (C), and quantitative analysis (D) for the percentage of apoptotic positive cells in kidneys from *Pkd1$^{fl/fl}$:Smyd3$^{+/+}$:Ksp-Cre* neonates, as detected by TUNEL assay. Scale bars: 20 μm.

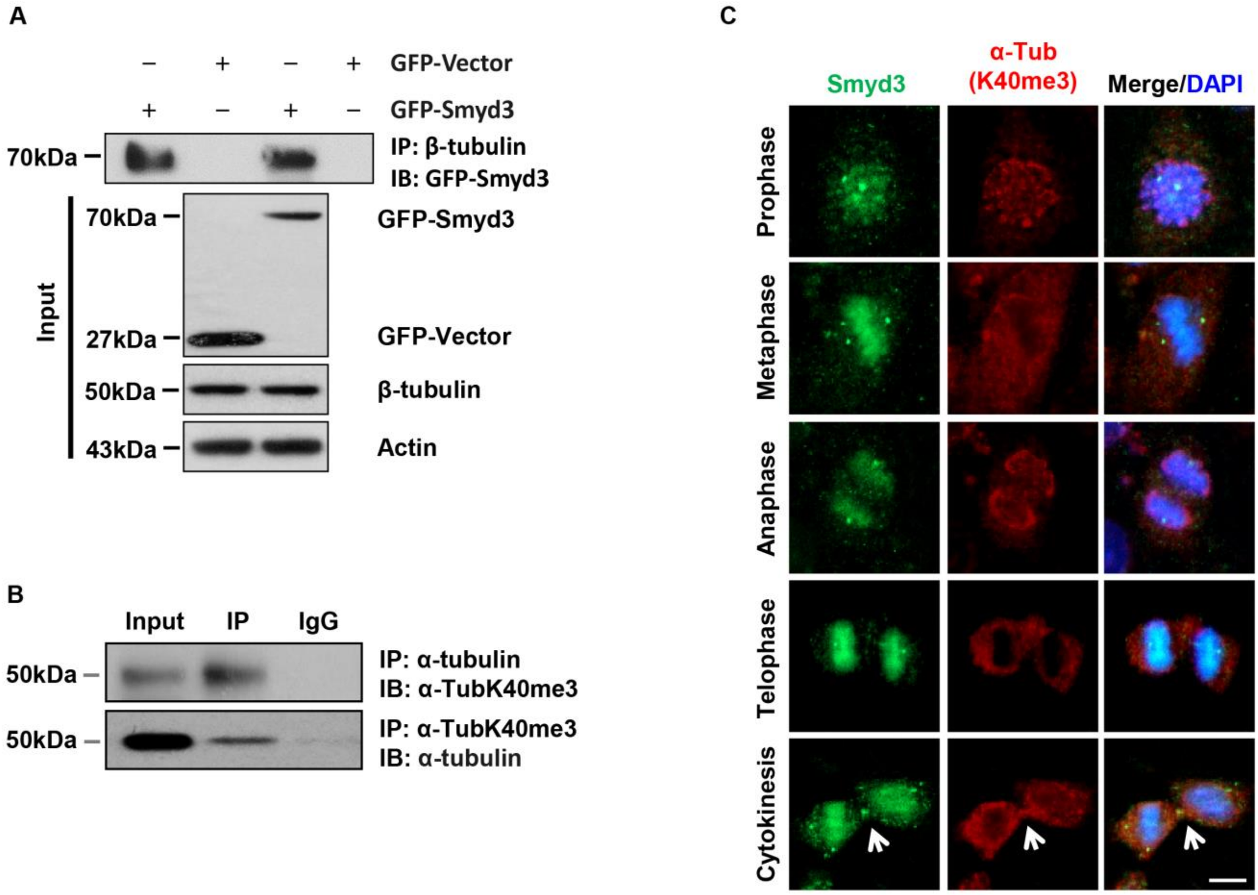

Supplemental Figure S3. SMYD3 forms a complex with α-tubulin and β-tubulin. (**A**) Co-immunoprecipitation analysis reveals the interaction between GFP-SMYD3 and β-tubulin in HEK293T cells, and endogenous SMYD3 and β-tubulin in PN24 cells. (**B**) Co-immunoprecipitation analysis reveals the interaction between α-tubulin and methylated tubulin at K40 in HEK293T cells. (**C**) SMYD3 co-localizes with methylated α-tubulin during cytokinesis. Representative images of IMCD3 cells co-stained with methylated α-tubulin antibody α-TubK40me3 (red), and Smyd3 (green). Images show co-localization of SMYD3 and α-TubK40me3 during cytokinesis. Scale bar, 20 μm.

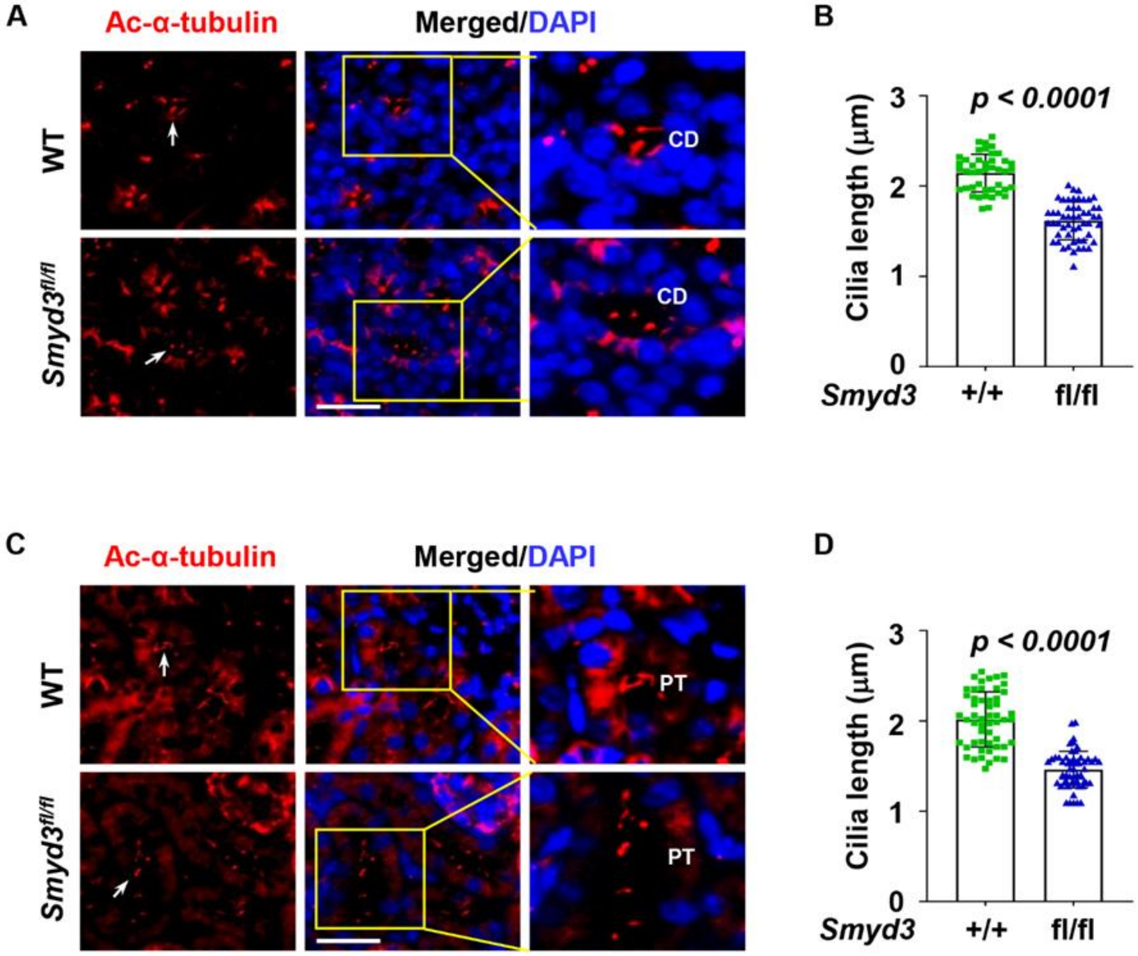

Supplemental Figure S4. Single conditional knockout of *SMYD3* inhibits ciliogenesis. (**A** – **D**) Smyd3 regulates ciliogenesis in kidney tissues. Paraffin-embedded kidney sections were immunostained with the ciliary marker acetylated α-tubulin (Ac-α-tub) and cilia are indicated by white arrows. Representative images (**A**), and quantitative data (**B**), of cilium length in kidney collecting ducts (CD) in *Pkd1*$^{+/+}$:*Smyd3*$^{+/+}$:*Ksp-Cre* (WT, n=45) and *Pkd1*$^{+/+}$:*Smyd3*$^{fl/fl}$:*Ksp-Cre* (*Smyd3*$^{fl/fl}$, n=45) kidneys at P7. Representative images (**C**) and quantitative data (**D**), of cilium length in kidney proximal tubules (PT) in *Pkd1*$^{+/+}$:*Smyd3*$^{+/+}$:*Ksp-Cre* (WT, n=40) and *Pkd1*$^{+/+}$:*Smyd3*$^{fl/fl}$:*Ksp-Cre* (Smyd3$^{fl/fl}$, n=50) kidneys at P7. The quantitative data of cilium lengths were measured from 5 to 10 regions for each mouse kidney, (n = 3). Scale bar, 20 μm.
